# Reviving Old Antibiotics: New Indications and Therapeutic Perspectives—A Review

**DOI:** 10.3390/ph19020278

**Published:** 2026-02-06

**Authors:** Paweł Radkowski, Julia Oszytko, Kamil Sobolewski, Florian Trachte, Maja Czerwińska-Rogowska, Dariusz Onichimowski, Marta Majewska

**Affiliations:** 1 Department of Anaesthesiology and Intensive Care, School of Medicine, Collegium Medicum, University of Warmia and Mazury in Olsztyn, 10-719 Olsztyn, Poland; dariusz.onichimowski@uwm.edu.pl; 2 Clinical Department of Anaesthesiology and Intensive Care, Regional Specialist Hospital in Olsztyn, 10-561 Olsztyn, Poland; kamil.roman.sobolewski@gmail.com; 3 Hospital zum Heiligen Geist, 34560 Fritzlar, Germany; 4 Faculty of Medicine, Opole University, 45-758 Opole, Poland; oszytkojulia@gmail.com; 5 Medical Sociology Unit, Hannover Medical School, 30625 Hannover, Germany; trachte.florian@mh-hannover.de; 6 Doctor’s Office, C. Zuleger Extertal, 32699 Extertal, Germany; 7 Department of Human Nutrition and Metabolomics, Pomeranian Medical University, 71-460 Szczecin, Poland; maja.czerwinska.rogowska@pum.edu.pl; 8 Department of Human Physiology and Pathophysiology, School of Medicine, Collegium Medicum, University of Warmia and Mazury in Olsztyn, 10-719 Olsztyn, Poland; marta.majewska@uwm.edu.pl

**Keywords:** drug-resistant infections, fosfomycin, colistin, streptomycin, vancomycin

## Abstract

The rapid global spread of antimicrobial resistance (AMR) has significantly reduced the effectiveness of many modern antibiotics, creating an urgent need for alternative therapeutic strategies. One promising approach is the revival and repurposing of older antimicrobial agents whose clinical potential was previously limited by toxicity concerns, pharmacokinetic challenges, or the availability of newer drugs. Recent advances in drug delivery, dosing optimization, and antimicrobial stewardship have renewed interest in these compounds as viable options for the treatment of multidrug-resistant infections. The aim of this review is to provide a comparative, clinically oriented analysis of selected “old” antibiotics, fosfomycin, colistin, streptomycin, and vancomycin, with emphasis on their current therapeutic roles, pharmacokinetic/pharmacodynamic (PK/PD) targets, toxicity mitigation strategies, resistance mechanisms, and evidence supporting combination therapies and alternative routes of administration. This narrative review was conducted using a structured PubMed search and manual reference screening, focusing on clinical, PK/PD, and translational studies relevant to the contemporary use of legacy antibiotics. The review summarises current evidence on the re-emerging clinical applications of these agents, each discussed in the context of historical use, mechanism of action, resistance patterns, and newly identified indications. Attention is given to novel formulations, combination strategies, and alternative routes of administration that enhance efficacy while limiting toxicity, including applications in biofilm-associated infections. Overall, strategic repurposing of older antibiotics represents a valuable complementary approach in the fight against AMR and may extend the therapeutic lifespan of existing agents in an era of limited antibiotic innovation.

## 1. Introduction

Antibiotics (a name derived from the Greek anti, meaning against, and bios, meaning life) have been a part of humanity for centuries, although the methods of their conscious use were unknown for a long time. The first known use of substances with antibiotic effects occurred in ancient Nubia, between 350 and 550 BC, when beer containing tetracycline was consumed. Although people at the time were unaware of antibiotics’ existence, they used them intuitively, likely because of their beneficial effects on health [1]. The official discovery of this group of drugs is attributed to Alexander Fleming, who described penicillin in 1928. For many years, they were used as a remedy for ailments, even in unjustified cases, which led to increasing resistance to them. The first strains of bacteria that were resistant began to be described in the mid-20th century. In the era of escalating antimicrobial resistance, there has been renewed clinical interest in the reintroduction of the first antibiotics, such as fosfomycin, colistin, streptomycin, and vancomycin [2].

Judicious patient selection and dosing optimisation are critical. Therapeutic drug monitoring may play a key role in maximising efficacy while limiting toxicity. Uncontrolled reintroduction risks recreating historical patterns of resistance. The biggest problem in their reuse is the gaps in the basic information necessary for their correct prescription. First, the dosing method is highly diverse, both in terms of doses and administration frequency, suggesting a lack of clearly defined therapeutic regimens. Second, many current clinical indications are based on outdated clinical data from the period of their original use, which may result in inadequate application in current clinical settings. Third, pharmacokinetics should be examined in special patient groups, such as obese, pediatric, or dialysis patients—these data are often incomplete or unavailable [3].

In addition to the need to verify the effectiveness of “old” antibiotics using modern research tools, it is also important to emphasise that technological advancements enable alternative routes of administration for these drugs beyond those used in the last century. This review provides a clinically oriented, evidence-based synthesis of pharmacokinetic, pharmacodynamic and therapeutic data supporting the reintroduction of selected legacy antibiotics in the era of multidrug-resistant infections. Special emphasis is placed on dosing optimisation, combination therapy, and alternative routes of administration relevant to multidrug-resistant and biofilm-associated infections.

Fosfomycin, colistin, streptomycin, and vancomycin were selected as representative agents from four distinct antibiotic classes that remain clinically relevant as rescue or adjunctive therapies in the management of multidrug-resistant infections. These include carbapenem-resistant Gram-negative pathogens, methicillin-resistant *Staphylococcus aureus* (MRSA), vancomycin-resistant *Enterococcus* (VRE), and drug-resistant tuberculosis. The contemporary clinical use of these antibiotics is frequently constrained by narrow therapeutic windows, substantial pharmacokinetic variability, and the need for dose optimisation and/or therapeutic drug monitoring. A specific aim of this review is to compare these agents within a clinically oriented framework that integrates pharmacokinetic/pharmacodynamic (PK/PD) targets, toxicity mitigation strategies, resistance mechanisms, and the available evidence supporting combination therapies and alternative routes of administration, with particular emphasis on biofilm-associated infections.

## 2. Materials and Methods

This review was developed as a narrative synthesis based on a structured literature search. We searched the PubMed database from 1 January 2000 to 15 January 2026, using a combination of controlled vocabulary and free-text terms for each antibiotic (‘fosfomy-cin’ ‘colistin’ OR ‘polymyxin E’ OR “streptomycin” and ‘vancomycin’) along with terms describing pharmacokinetics, pharmacodynamics, dosing, therapeutic drug monitoring, toxicity, resistance, biofilm, and combination therapy. In addition, we conducted a target-ed manual search of key guideline documents and PK/PD studies identified from refer-ence lists. We prioritised systematic reviews, clinical pharmacokinetic/pharmacodynamic studies, clinical trials, and observational outcome studies. We included preclinical in vitro and animal studies if they addressed mechanistic issues (e.g., resistance mechanisms) or demonstrated clinically plausible combination or alternative strategies. For combination regimens, we classified supporting evidence as follows: (i) in vitro studies (including bio-film models), (ii) animal models, (iii) clinical case reports/case series, or (iv) controlled clinical trials (randomised or non-randomised). The levels of evidence are listed in the text and summarised in Table 1, placed at the end of the manuscript to serve as an overarching comparative summary.

## 3. Results

### 3.1. Fosfomycin (FOS)

Fosfomycin (1,2-epoxy-propyl-phosphonic acid) is an antibiotic with a unique structure, unrelated to any other known group of antibiotics. It was discovered in 1969 by a 14-person Spanish-American research group. In a soil sample from the Alicante mountains, the bacterium *Streptomyces fradiae* was isolated—a natural producer of the antibiotic fosfomycin, which can also be synthesised by *Streptomyces viridochromogenes* and *Streptomyces wedmorensis* [4]. It is available in the form of disodium salt used orally and parenterally, or for oral use only, calcium salt or from tris-hydroxymethyl-aminomethane (tromethamine), with an average half-life of 5.7 h [4,5,6,7]. The addition of tromethamine increases bioavailability to 34–58%, compared with only 12% for the calcium salt after oral administration [8].

Despite its low molecular weight (138.059 g/mol), one of the weakest among antibiotics, its high hydrophilicity, and its low degree of protein binding, it diffuses well into tissues [6,9]. Fosfomycin is an analogue of phosphoenolpyruvate (PEP) having a phosphonic group and an epoxy ring in its structure. Its action occurs during the bacterial growth phase by covalently binding to the enzyme MurA (N-acetylglucosaminylpyruvate transferase), thereby inhibiting the initial stage of cell wall synthesis and causing lysis of both Gram-positive and Gram-negative bacteria [4,7]. The drug enters their interior thanks to the glycerol-3-phosphate (GlpT) and glucose-6-phosphate [G6P] (UhpT) transporters [6]. Fosfomycin has been the “gold standard” for the treatment of uncomplicated cystitis in women since its approval. The advantage of its use is the possibility of administering a single dose of the drug [4,10,11], as well as its high tissue penetration, including the cerebrospinal fluid, abscesses, and bones [12]. In recent years, the popularity of this drug has increased significantly due to its use as an alternative to mono- or polytherapy, driven by the emergence of multidrug-resistant bacterial strains [5,12]. It was used in cases involving critical and high-priority organisms, including MRSA (methicillin-resistant *Staphylococcus aureus*), CRE (carbapenem-resistant Enterobacterales), VRE (vancomycin-resistant Enterococci), MRCNS (methicillin-resistant coagulase-negative Staphylococci), or *Pseudomonas aeruginosa* [7,13]. Despite its similar effect to beta-lactams, fosfomycin exhibits a low level of cross-resistance induction [13,14]. Despite its presence on the market for over fifty years, *E. coli* resistance is rarely reported, in contrast to *Acinetobacter baumannii*, which has developed defence mechanisms in most strains [6,15]. FOS-targeted antibiotic resistance models can be divided into two categories: those based on the site of changes (plasmid or chromosomal) and those based on the three possible effects obtained:

#### 3.1.1. Reduced Uptake (GlpT/UhpT Transporters and Regulators)

Chromosomal changes are more common, involving modifications to the genes encoding the GlpT and UhpT transporters or to sequences containing information about their regulators. Both methods disrupt drug penetration into the cell. This has been primarily described in the cases of *Escherichia coli* and *Pseudomonas aeruginosa* [7,16,17]. Disturbances in the chromosomal sequence of the Abrp gene, which is responsible for proper bacterial growth and cell membrane integration, can also lead to loss of sensitivity. This mutation is characteristic of *Acinetobacter baumannii* [6].

#### 3.1.2. Target Modification (MurA)

Another developed method of intrinsic resistance is changing the target of the antibiotic’s action. This occurs by changing the amino acid cysteine to aspartate in the active site of MurA, which prevents FOS from binding. *Mycobacterium tuberculosis* naturally possesses this mechanism, since the structural change occurs at position 117 of the MurA enzyme [7,18].

#### 3.1.3. Enzymatic Inactivation (Fos Enzymes and Kinases)

A mechanism that may pose a serious challenge to modern medicine is antibiotic inactivation. Multiple metalloenzymes are responsible for this. FosX, together with FosA, incorporates a water molecule and a tripeptide made of glutamic acid, glycine, and L-cysteine-glutathione into the epoxide ring structure. FosB adds cysteine or a bacillithol molecule. The neutralisation effect can also be achieved by phosphorylation of the antibiotic’s phosphate groups, catalysed by FomA and FomB kinases, resulting in the formation of diphosphate and triphosphate molecules that lack bactericidal properties [7].

Currently, fosfomycin is experiencing a resurgence due to its broad-spectrum activity and the search for new methods to eradicate multidrug-resistant pathogens. For years, it has been used to treat urinary tract infections and is now increasingly used to treat acute bacterial prostatitis (ABP) and chronic prostatitis (CP). The National Institute of Health distinguishes the division into categories I and II, where treatment is complex due to high resistance and limited distribution into the glandular tissue [19]. The use of fosfomycin tromethamine, with its low molecular weight, high hydrophilicity, low protein binding, and renal excretion in a practically unmodified form, results in high bioavailability (33–50%). 2 h after administration of a 3 g dose, a concentration of 2–2.5 g is obtained in urine [20]. Studies have shown that oral administration limits inflammation, reduces bacterial proliferation, and decreases the degree of glandular structure destruction [21]. Bacteria exhibit various minimum inhibitory concentrations (MICs); therefore, some researchers do not recommend administering fosfomycin when the MIC exceeds 4 mg/L. This is due to the possibility of not achieving non-therapeutic concentrations [22]. Numerous studies have evaluated the efficacy of this drug in treating CP. Marino et al. analysed 81 cases, in which *Escherichia coli* was identified as the predominant pathogen (59/81, including 3 ESBL-producing strains). Less frequently, infections were caused by *Klebsiella pneumoniae* (n = 8), *Enterococcus faecalis* (n = 6), *Klebsiella oxytoca* (n = 4), *Proteus mirabilis* (n = 2), *Pseudomonas aeruginosa* (n = 1), and *Raoultella planticola* (n = 1). Patients were given 3 g for 24–72 h for 5–13 weeks. The dosing regimen varied between studies (maintaining the same dose throughout the treatment period or reducing the frequency of drug administration to 2 or 3 times after 7–9 days of initiating antibiotic therapy, with a daily dose). Still, in most cases, clinical improvement and pathogen eradication were achieved [6]. In the case of ABP, fosfomycin also demonstrates a favourable action profile, although it is not a first-line drug. Therapeutic success was achieved in two independent cases of *E. coli* ESBL infections. In a 30-year-old man, the antibiotic was started at a dose of 3 g/24 h for the first week, and then the frequency of administration was reduced to 3 g/48 h for two weeks [6], and in the case of a 73-year-old man, the therapy was administered at a dose of 3 g daily for 16 weeks [23]. In the treatment of *E. faecalis* infection resistant to ampicillin, chloramphenicol, vancomycin, gentamicin and ciprofloxacin, FOS was administered at a dose of 3 g every 72 h for 3 weeks [24]. Some researchers recommend using FOS when ABP is caused by organisms resistant to quinolones [25]. Delgado et al. also emphasise the use of fosfomycin in the treatment of endocarditis [26]. A review by Tozluyurt et al. collected 64 *E. coli* strains from different sources; all were found to be resistant to Aztreonam and Aztreonam–avibactam according to EUCAST guidelines, but all were susceptible to FOS. Resistance to FOS worldwide remains low—less than 10% [27]. Falagas et al. analysed in vitro data showing that the sensitivity of *E. coli* with developed ESBL resistance to FOS was 96.8%, and that of *K. pneumoniae* was 81.3% [11]. Multidrug-resistant strains of *P. aeruginosa* were examined, and 61% were found to be susceptible to FOS [28]. Bodmann et al. analysed 716 cases of severe bacterial infections of various etiologist from 5 European countries (34.6% of pathogens were multidrug-resistant), of which 41.6% developed sepsis or septic shock. In all cases, the pathogen was sensitive to FOS. It was noted that treatment with a high-dose regimen, i.e., >16 g intravenously per day, brought significantly better results than in patients receiving lower doses [13]. Due to its mechanism of action, which inhibits cell wall synthesis, it is used synergistically with many antibiotics to enhance therapeutic efficacy. Usually, in the case of *Enterobacterales* eradication, it is used in combination with penicillin (51%), carbapenems (43%), chloramphenicol (39%) and cephalosporin (33%) [27,29].

The combination of antibiotics, particularly in the treatment of biofilm-associated infections, has gained substantial attention in antimicrobial research. Biofilms, communities of bacteria encased in a protective extracellular matrix, are notoriously difficult to treat due to their inherent resistance to standard antibiotic therapies. As antibiotic resistance continues to rise globally, the development of highly effective antibiotic combinations is crucial for enhancing patient outcomes and addressing this pressing health crisis [30,31].

Combination therapy is frequently considered for biofilm-associated infections and for infections caused by multidrug-resistant Gram-negative pathogens. Wang et al. reported enhanced activity of fosfomycin combined with ciprofloxacin and gentamicin against biofilms formed by *Escherichia coli* and *Pseudomonas aeruginosa* (in vitro biofilm model) [32]. Fosfomycin has also demonstrated antibiofilm effects that may facilitate penetration into established biofilm matrices (predominantly preclinical evidence) [33,34].

For infections due to carbapenemase-producing *Enterobacterales*, fosfomycin is commonly incorporated into combination regimens with colistin, carbapenems (e.g., imipenem, ertapenem) or tigecycline; the supporting clinical evidence is largely derived from case series and observational data, and regimen choice should be individualised to susceptibility profiles and infection site [7]. A promising approach for *Pseudomonas* aeruginosa infections in cystic fibrosis is fosfomycin combined with fluoroquinolones or aminoglycosides, for which synergistic activity has been reported predominantly in in vitro models [35]. Finally, co-administration of fosfomycin with foscarnet, a FosA inhibitor, markedly increased intracellular fosfomycin concentrations and improved antibacterial activity *against Pseudomonas aeruginosa*, *Klebsiella pneumoniae* and *Enterobacter cloacae* in vitro, supporting an enzyme-targeted potentiation strategy [36].

### 3.2. Colistin (COL)

Colistin (also known as polymyxin E) is a polymyxin antibiotic, discovered by Y. Koyama in 1947 as a metabolite of *Paenibacillus polymyxa* subsp. *colistinus* found in soil [37]. Until the 1980s, it was the first-line drug for treating infections caused by Gram-negative bacteria; however, it was abandoned due to the rapid emergence of alternative pharmacotherapies with a lower risk of neurotoxicity and nephrotoxicity. It regained popularity in the 21st century for use against multidrug-resistant microorganisms [38,39]. Between 1990 and 2000, it was used to treat lung infections caused by multidrug-resistant bacteria in patients with cystic fibrosis [40]. Recently, its use has increased rapidly as it is a drug of last resort in critical conditions [35]. Initially administered topically to the eyes and ears, later intravenously in cases of infectious diarrhoea and urinary tract infections [41,42]. Currently, it is administered intravenously at a dose of 300 mg, with a 10% dose increase recommended for dialysis patients due to potential drug loss during dialysis [43,44]. According to Haseeb et al., a dose of 2.5–5 mg/kg/day was usually recommended in antibacterial therapy [45].

From a pharmacodynamic perspective, colistin displays rapid, concentration-dependent bactericidal activity. This occurs by binding to lipopolysaccharide on the outer membranes of Gram-negative bacteria, displacing divalent cations (Mg^2+^, Ca^2+^), disrupting membrane integrity, and facilitating easy penetration through the inner membrane. The most effective way to describe colistin’s efficacy is by the AUC:MIC ratio (and unbound fAUC:MIC for pulmonary delivery) as the primary pharmacokinetic/pharmacodynamic index. Additionally, penetration into the cell leads to an increased synthesis of reactive oxygen species (ROS), which damages the structures necessary for its functioning. As a consequence of these processes, it dies [38,44]. Researchers also provide alternative mechanisms of drug action, including the disruption of both cell membranes due to phospholipid exchange [42] and the inhibition of lipid A endotoxin activity, leading to neutralisation of lipopolysaccharide (LPS) [46,47,48]. The antibiotic is available in two forms—the prodrug, colistin methanesulfonate (CMS), can be administered intravenously, intrathecally, intraventricularly, or by nebulization [41,49]. However, 20–25% of the dose is metabolised to the active form, which is why the time to reach a therapeutic dose of the drug usually exceeds 36 h [50]. The more toxic form, the sulfate salt, is intended for oral use to eradicate the gastrointestinal tract and for local application on affected skin. Systemic administration is typically avoided due to the high risk of nephrotoxicity and bronchospasm [51,52]. In general, adverse effects are reversible upon discontinuation of treatment [53]. Intravenously, colistin is administered as the inactive prodrug CMS, which undergoes slow and variable hydrolysis in plasma to form the active drug, resulting in highly variable pharmacokinetics characterised by poor tissue penetration due to its large, cationic peptide structure, predominantly extracellular distribution, and renal elimination that necessitates loading doses (typically 9 million IU) followed by maintenance dosing adjusted for renal function to achieve therapeutic plasma concentrations within a narrow therapeutic window [54,55]. When administered via inhalation or nebulisation, CMS achieves significantly higher local lung concentrations, with minimal systemic exposure. This results in epithelial lining fluid (ELF) colistin concentrations that are substantially higher than concurrent plasma levels (with only ~9% of the aerosol dose reaching ELF) yet still achieving improved local fAUC/MIC targets. This route is particularly valuable for treating ventilator-associated pneumonia and cystic fibrosis lung infections, while minimising the risk of nephrotoxicity [56,57,58]. Oral administration of colistin sulfate has been found to exhibit poor gastrointestinal absorption. As a result, it is primarily used for selective intestinal decontamination rather than systemic therapy. However, topical formulations, including newer nanoemulsion and neoemulgel preparations, have been developed to enhance skin permeation for localised antimicrobial activity in wound infections [54,59,60]. Intrathecal or intraventricular administration has been reported as a last-resort approach for central nervous system (CNS) infections when systemic dosing fails to achieve adequate CNS penetration. However, there is a paucity of standardised dosing protocols and detailed pharmacokinetic data for this route [59].

Currently, its primary use is limited to sepsis, severe bacteremia, and pneumonia in mechanically ventilated patients [40]. Nebulisation is an alternative route of colistin administration that allows achieving therapeutic concentrations in the alveolar space without systemic effects, thus reducing the likelihood of adverse effects [61,62]. An analysis was conducted comparing intravenous colistin therapy alone and intravenous colistin therapy together with nebulisation. A higher clinical response rate was achieved, with no nephrotoxicity and reduced mortality with combined treatment [63]. The results of a separate study indicate better clinical outcomes, but without an impact on mortality when colistin nebulisation was added to antibiotic therapy [64]. It should be noted that, due to the high level of drug binding to mucin (approx. 85%), inhaled antibiotic therapy should be combined with another active antibiotic [44]. In recent years, polymyxin E has become the first-line drug for eradicating multidrug-resistant bacteria, especially *Enterobacterales* producing carbapenemases [65,66]. A definite advantage of its implementation in treatment is the difficulty in acquiring resistance and the ease of losing it in the absence of contact with the antibiotic [39].

Most often, bacteria acquire resistance to colistin through chromosomal mutations in genes that modify lipid A, a building block of LPS [39]. This occurs because of the addition of phosphoethanolamine (PEtN) and 4-amino-4-deoxy-L-arabinose (L-Ara4N) to the phosphate groups of lipid A [38]. In 2016, a new mechanism for generating resistance in microorganisms was described: the plasmid-mediated mcr-1 gene. Its mode of action is based on neutralising the negative charge of LPS and on its inability to interact with the antibiotic [61]. The use of efflux pumps and the synthesis of the capsule are also described [38]. Colistin is classified as a narrow-spectrum antibiotic. It does not affect anaerobic bacteria, and the species naturally resistant to it include *Proteus* spp., *Edwardsiella* spp., *Providencia* spp., *Aeromonas* spp., *Chromobacterium* spp., *Brucella*, *Morganella morganii*, *Vibrio cholerae*, *Burkholderia cepacia*, *Legionella*, *Burkholderia mallei*, *Campylobacter*, and *Serratia marcescens* [40,51].

Volkow-Fernández et al. described a case of chronic urinary tract infection caused by *Acinetobacter baumannii*, in which intravesical infusion was used, resulting in eradication after 10 days of treatment. It is worth emphasising that no side effects occurred [67]. Due to the low likelihood of colistin penetrating the blood–brain barrier, intrathecal administration is permitted in cases of meningitis. The review included 64 cases, of which 80% achieved therapeutic success. Meningeal irritation occurred in 12 patients [68].

Because colistin is often deployed as a last-line agent in severe infections and resistance can emerge during therapy, it is frequently used in combination with other antibiotics. The strongest evidence for synergistic activity comes from in vitro studies, including post-antibiotic effect and synergy assays against colistin-resistant KPC-producing *Klebsiella pneumoniae* and other multidrug-resistant Gram-negative pathogens [69]. Diani et al. reported high efficacy of colistin–meropenem combination therapy [70]. Similarly, combinations of polymyxin E with carbapenems, tigecycline, or rifampicin demon-strated superior outcomes compared with monotherapy in infections caused by car-bapenemase-producing *Klebsiella pneumoniae* [40]. In these pathogens, colistin has also been used in combination with fosfomycin, where disruption of the bacterial outer membrane by colistin facilitates greater intracellular penetration of fosfomycin. However, the magnitude of this synergistic effect appears to vary across bacterial strains [7]. In *Pseudomonas* spp. infections, colistin-based polytherapy with rifampicin, ceftazidime, or amikacin has also shown favourable activity [40].

### 3.3. Streptomycin (STR)

Streptomycin, the first antibiotic from the aminoglycoside group to be discovered, was isolated in 1944 by Selman Waksman and his student Albert Schatz from the actinomycete *Streptomyces griseus*. For this achievement, Waksman received the Nobel Prize 8 years later [1]. Initially, it was used against Gram-positive and Gram-negative bacteria, but over time, studies showed its potential against Gram-positive cocci. Ultimately, it gained popularity as a means of combating tuberculosis bacilli [13]. Initially, it was used as monotherapy, but after a few years, it was combined with isoniazid and para-aminosalicylic acid; however, even this combination did not prevent the development of resistance [71,72]. Since 1991, its use has been considered an additional treatment, but as microorganisms have become increasingly resistant to other antibiotics, it is gaining popularity again [73]. It is used as a substitute for amikacin and in situations where it is not possible to purchase more expensive substances [74].

#### Contemporary Clinical Use: PK/PD Considerations and Resistance

Streptomycin is primarily used as an injectable second-line or adjunctive agent for tuberculosis. Modern guidelines recommend weight-based dosing, with an initial daily period followed by reduced frequency. As an aminoglycoside, efficacy is exposure-dependent and is most closely associated with peak concentration-to-MIC (Cmax/MIC) and, in some settings, AUC/MIC targets. Adults should receive a daily dose of 15 mg/kg i.m. (with a maximum of 1 g/day). This is to be taken as a single initial dose, followed by 3 doses of 15 mg/kg per week [75,76]. Historical treatment regimens for miliary and menin-geal tuberculosis have been reported to include much higher daily doses (ranging from 20 mg per pound to 44 mg/kg and up to 2 g i.m. daily) and the use of intrathecal streptomycin (50–100 mg intrathecally for tuberculous meningitis). However, these higher historical doses have been associated with toxicity and are not the standard recommendation for modern treatment [77,78].

Due to the evolutionary similarity between human and bacterial mitochondria, the drug can bind to them, potentially leading to side effects such as ototoxicity [79]. At the bacterial ribosome, streptomycin binds irreversibly to the 30S subunit, with key interactions involving 16S rRNA and the S12 protein, leading to misreading and inhibition of protein synthesis [80,81]. Reported streptomycin resistance among *M. tuberculosis* isolates remains substantial in several settings (e.g., 82.7% in the Porto region in 2007–2013; 64.9% in a Chinese cohort) [82,83]. Resistance most commonly arises through mutations in rpsL, rrs and gid, which affect the S12 protein or 16S rRNA and alter drug binding; suboptimal regimens and prolonged exposure without appropriate companion drugs are recognised drivers of resistance selection [84,85]. Morphological changes, such as atrophy of the crests and swelling of the organelle, are described. Streptomycin inhibits COX-1, impairing cytochrome c oxidase function and increasing ROS production. The participation of these molecules allows us to distinguish this type of cell death from ferroptosis, although both are dependent on iron [77]. Its mode of action involves irreversible binding to the small ribosomal subunit, preventing the proper synthesis of peptides and proteins necessary for the bacterial cell’s functioning and survival. The aim of the action is the interaction between regions located within 16S rRNA and the K45 residue present on the S12 protein [80,81]. *M. tuberculosis* resistance to STR in the years 2007–2013 in the Porto area was 82.7% [82,83], while in a study conducted in China, it was 64.9% of cases [18]. While the precise mechanisms of resistance acquisition are not completely elucidated, it is widely accepted that long-term, extensive use of antibiotics without appropriate combinations and suboptimal dosing contributes significantly to its development [84,85]. One of the main theories is that a mutation occurs within the rpsL, rrs, and gid genes, which encode the S12 protein and 16S rRNA. There is also a hypothesis that bacterial homocysteine synthase, an essential enzyme in the methionine synthesis pathway, initiates resistance [84]. Beyond established antibacterial indications, streptomycin has been explored for non-antibacterial applications (e.g., anticancer and antiviral concepts); however, the available evidence is predominantly preclinical and should be interpreted as hypothesis-generating rather than supporting clinical use. Karp and Lyakhovich report that this antibiotic impairs mitochondrial function, thereby disrupting oxidative phosphorylation, which is necessary for cancer cell growth [86]. Itoh et al. focused on understanding the mechanism that could be key in designing targeted therapy. They demonstrated that hydration of one of the drug’s aldehyde groups results in the formation of a diol, which enables integration into the ribosomal RNA backbone [87]. Guillorit et al. investigated the targeted action of STR on cells that initiate colon and breast cancer, which are responsible for metastasis. Administration of the antibiotic inhibited “sphere nucleation” but not “sphere growth.” In an animal model, this effect translated into suppression of metastatic initiation. This property appeared to be specific to spectinomycin, as none of the other aminoglycosides tested showed similar activity [80].

In parallel with the search for new therapeutic applications, innovative delivery systems for STR are also being explored. Karimitabar et al. evaluated solid lipid nanoparticles loaded with STR for the treatment of brucellosis, which is mostly caused in humans by *Brucella melitensis* and *Brucella abortus*. A key advantage of this formulation was sustained release, approximately 80% of the drug was released within 100 h, together with efficient uptake by macrophages, without any observed loss of stability or increased cytotoxicity [88]. Guerra et al. investigated pullulan nanofibers impregnated with STR in rats, demonstrating effective antimicrobial activity, prolonged release, and targeted delivery. The capsules dissolved in the small intestine, and thanks to the mucoadhesive properties of the nanoparticles, were subsequently transported to the large intestine [89]. STR combined with nanomedicine has also been evaluated in sepsis therapy. In a study by Wei and Ma, the peptide GF9, which inhibits the secretion of inflammatory mediators from myeloid cells via interactions with the TREM-1 receptor, was co-administered with streptomycin, resulting in enhanced anti-inflammatory and bactericidal effects and improved survival in an animal model [90]. The use of this protein, combined with STR, demonstrated strong anti-inflammatory and bactericidal effects, resulting in a higher survival rate than in the control group [84]. The use of nanocarriers in streptomycin therapy enables controlled, targeted drug release and increased bioavailability without increased cytotoxicity. The observed antimicrobial and anti-inflammatory effects in brucellosis and sepsis models indicate high efficacy of such forms of administration. Further studies are needed on their use in the clinic and extension to other disease entities.

### 3.4. Vancomycin (VAN)

Vancomycin is a first-generation glycopeptide antibiotic of natural origin, synthesised by the Actinobacterium *Amycolatopsis orientalis*. It was introduced in 1958 following its discovery by researchers at Eli Lilly in the United States [91,92]. It has a wide range of applications but is primarily used for infections caused by Gram-positive bacteria, including MRSA [93]. In the review by Elrggal et al., a divided dosing regimen is recommended, with dosing every 6–8 h. The loading dose should be 20–25 mg/kg, followed by a maintenance dose of 15–25 mg/kg. In patients, this antibiotic dose is subtherapeutic; therefore, the recommended dose is 40–60 mg/kg/day [94]. It is particularly recommended for the treatment of brain abscesses [95] and skeletal system infections [96,97]. The mechanism of action of VAN is based on the inhibition of the synthesis of components necessary for cell wall synthesis—N-acetylmuramic acid and N-acetylglucosamine polymers. This occurs as the drug binds to the D-Ala-D-Ala part of lipid II [92,98]. The integrity of the cell wall is compromised, leading to rupture and subsequent cell lysis.

From a PK/PD perspective, vancomycin efficacy in serious MRSA infections is best predicted by the 24 h area under the concentration-time curve to MIC ratio (AUC/MIC). Contemporary consensus guidelines recommend targeting an AUC/MIC of 400–600 (assuming an MIC of 1 mg/L by broth microdilution) to maximise efficacy while limiting nephrotoxicity; AUC-guided monitoring is preferred over trough-only strategies [99].

The first organisms resistant to VAN were isolated in 1986 from one of the *Enterococcus* species [100]. The mechanism of its occurrence can be divided into two ways: changing the amino acid sequence within lipid II from D-Ala-D-Ala to D-Ala-D-lac or to D-Ala-D-Ser [92]. Due to adverse effects mainly affecting the kidneys or hearing organs, researchers are seeking alternative methods of antibiotic administration to minimise them [95,101]. Florczyk et al. propose using chitosan particles in combination with an antibiotic embedded in hydrogels, thereby delivering the drug to the desired site while avoiding systemic effects. Additionally, the supply of the drug can be strictly controlled, which reduces the risk of resistance. Another advantage is the rapid attainment of high concentration with simultaneous prolonged release of VAN [101].

One known indication for the use of the first glycopeptide antibiotic is MRSA endocarditis [102]. Save et al. in their study focused on optimising treatment using bacteriophages to reduce the dose of the administered antibacterial agent. For this purpose, an animal model was used, demonstrating that the method’s synergism allows for achieving better results than with single treatment. The use of this method in humans requires further studies and a personalised approach to each case, but it offers a good prognosis in the face of decreasing sensitivity to VAN [103].

Furthermore, Singh et al. observed an association between intestinal microbiota and the risk of hepatocellular carcinoma. VAN was used to eradicate bacteria with oncogenes on their surface, including LPS and lipoteichoic acid. In the treatment group, no neoplastic foci were found in the liver, and the treatment reduced macromolecular fatty liver disease. This effect on the human body has not been confirmed and, due to potential side effects and the risk of increased resistance, is controversial; however, these results may serve as an essential starting point for further research on therapies that modulate the intestinal microbiota in the context of cancer prevention [88].

### 3.5. Antibiofilm Strategies

Across biofilm-associated infections, the four agents reviewed here demonstrate complementary interactions that enhance penetration, suppress persisters, and improve killing within the extracellular matrix. In Gram-negative biofilms, colistin’s outer-membrane permeabilisation can potentiate partner drugs; chelating adjuvants such as EDTA further destabilise the matrix and lipopolysaccharide, restoring activity against catheter-associated, colistin-resistant *Klebsiella pneumoniae* biofilms in vitro and in vivo [104]. For *Pseudomonas aeruginosa*, azithromycin’s quorum-sensing and immunomodulatory effects, when paired with colistin or fluoroquinolones, accelerated clearance of established biofilms in a murine model despite limited in vitro synergy [105]. In Gram-positive biofilms, fosfomycin augments multiple anti-staphylococcal agents—linezolid, minocycline, vancomycin and teicoplanin—and the linezolid + fosfomycin combination reduced bacterial burden in a catheter-related MRSA biofilm rat model [106,107].

In implant-associated osteomyelitis, daptomycin + fosfomycin achieved superior biofilm eradication of MRSA compared with monotherapy in rats [108]. For device-related *Staphylococcus aureus* infection, vancomycin + rifampicin outperformed vancomycin alone in a prosthetic-joint mouse model, consistent with rifampicin’s antibiofilm activity and intracellular penetration, although some in vitro work cautions that certain rifampicin or gentamicin combinations can transiently delay early killing [109,110].

Finally, in enterococcal vegetations (biofilm-like structures), cell-wall–active agents (penicillin/ampicillin or vancomycin) increase aminoglycoside uptake and yield bactericidal synergy with streptomycin when high-level aminoglycoside resistance is absent [111].

**Table 1:** Comparative summary of fosfomycin, colistin, streptomycin and vancomycin (PK/PD, dosing considerations, resistance mechanisms, toxicity and common combination strategies, with evidence tier).

Antibiotic	PK/PD Index and Key PK Features	Dosing Considerations (Adult; Key Adjustments)	TDM/Monitoring	Key Toxicities	Resistance & Common Combinations (Evidence Tier)	Common Combinations (Evidence Tier)
Fosfomycin	Primarily AUC/MIC-driven exposure; low protein binding; wide tissue distribution [12,20].	Uncomplicated cystitis: 3 g PO single dose. Severe systemic infections: high-dose IV regimens (often >16 g/day) reported in real-world data. Renal adjustment required [13,20].	No routine TDM; consider exposure optimisation in critically ill where available. Monitor renal function/electrolytes.	Generally well tolerated; IV high-dose: sodium load, hypokalaemia. GI effects with oral formulations.	Resistance: reduced uptake (GlpT/UhpT), target alteration (MurA), enzymatic inactivation (FosA/FosB/FosX).	With beta-lactams/carbapenems for MDR Enterobacterales (clinical observational/case series); with aminoglycosides/fluoroquinolones for biofilm models (in vitro).
Colistin (polymyxin E)	Rapid concentration-dependent killing; primary index fAUC/MIC. Administered IV as colistimethate (CMS) prodrug with slow conversion [43,54].	IV CMS: loading dose + maintenance adjusted for renal function/RRT. Inhaled CMS: high local ELF exposure; consider adjunct systemic agent [43,56,57,58].	No widely standardised TDM; monitor renal function; avoid concomitant nephrotoxins where possible.	Nephrotoxicity; neurotoxicity; bronchospasm with inhalation.	Resistance: lipid A modification (chromosomal) and plasmid-mediated mcr genes.	With meropenem/carbapenems or tigecycline for CRE (clinical observational; in vitro synergy); IV + inhaled CMS for VAP (meta-analysis/observational
Streptomycin	Aminoglycoside: concentration-dependent killing; efficacy linked to Cmax/MIC (and AUC/MIC). Poor CNS penetration systemically [81].	TB and selected zoonoses: weight-based IM dosing; intermittent regimens reduce toxicity. Example TB regimens include 15 mg/kg daily or 25 mg/kg three times weekly in clinical studies [78].	Therapeutic drug monitoring used in some TB/NTM programs to target peaks and limit toxicity (setting-dependent).	Ototoxicity (vestibular/cochlear); nephrotoxicity; neuromuscular blockade (rare).	Resistance: rpsL/rrs/gid mutations; reduced uptake/enzymatic modification (aminoglycoside-class). Combinations: with cell-wall	With cell-wall active agents for enterococcal endocarditis when no high-level aminoglycoside resistance (clinical). Emerging anticancer/antiviral signals are preclinical only
Vancomycin	Time-dependent; target AUC/MIC 400–600 (assuming MIC = 1 mg/L) in serious MRSA [99].	Loading 20–25 mg/kg then AUC-guided maintenance; adjust for renal function/RRT; consider obesity/critical illness [94,99].	AUC-guided TDM recommended for serious MRSA infections; monitor renal function; consider hearing in prolonged courses.	Nephrotoxicity; infusion-related reactions (“red man”); rare ototoxicity.	Resistance: altered target (D-Ala-D-Lac/Ser in enterococci); VISA/VRSA mechanisms.	Rifampicin for device-related biofilm (animal/in vitro; selected clinical contexts); bacteriophage adjuncts remain preclinical/early translational

## 4. Discussion and Conclusions

In the context of escalating antimicrobial resistance, there has been renewed clinical interest in the reintroduction of “old” antibiotics, such as fosfomycin, colistin, streptomycin, and vancomycin. The reintroduction of older antibiotics should be embedded within antimicrobial stewardship programmes to ensure rational use, appropriate patient selection, and prevention of renewed resistance. Despite their long-term use, these drugs remain highly effective against multidrug-resistant microorganisms, especially in hospital infections, sepsis, and urinary tract and respiratory infections. Their judicious use may expand therapeutic options against multidrug-resistant pathogens while balancing efficacy, toxicity and resistance prevention. However, their reintroduction into medical practice requires updating pharmacokinetic and pharmacodynamic data, especially in patients in special groups, such as dialysis and pediatric patients or those with obesity. This creates clinically relevant knowledge gaps that complicate dosing decisions and highlight the need for individualised therapy supported by therapeutic drug monitoring. Modern treatment strategies, based on combination therapy and route-of-administration modification (e.g., nebulisation, nanocoated carriers), enable greater clinical efficacy while limiting toxicity and minimising the risk of resistance. As science advances, topics of its innovative use in oncology and antiviral therapy are emerging and being discussed. The key remains individualising therapy and monitoring therapeutic concentrations, especially in cases where drugs have a narrow therapeutic index, such as colistin and vancomycin. Reintegrating these antibiotics into modern medical practice not only expands the range of available therapeutic options but also offers hope for effective treatment of the most challenging cases of bacterial infections in the era of the antibiotic crisis. Future research should prioritise well-designed clinical studies addressing optimal dosing, combination regimens and novel delivery systems in high-risk patient populations.

## Data Availability

No new data were created or analyzed in this study. Data sharing is not applicable to this article.

## Abbreviations

The following abbreviations are used in this manuscript:

ABP	acute bacterial prostatitis
CMS	colistin methanesulfonate
CNS	central nervous system
COL	colistin (polymyxin E)
CP	chronic prostatitis
CRE	carbapenem-resistant *Enterobacterales*
ESBL	extended-spectrum β-lactamase
EUCAST	European Committee on Antimicrobial Susceptibility Testing
FOS	fosfomycin
FosA	fosfomycin resistance enzyme (glutathione transferase)
FosB	fosfomycin resistance enzyme (bacillithiol transferase)
FosX	fosfomycin resistance enzyme (epoxide hydrolase)
GlpT	glycerol-3-phosphate transporter
G6P	glucose-6-phosphate
L-Ara4N	4-amino-4-deoxy-L-arabinose
LPS	lipopolysaccharide
MIC	minimum inhibitory concentration
MDR	multidrug-resistant
MRSA	methicillin-resistant *Staphylococcus aureus*
MRCNS	methicillin-resistant coagulase-negative staphylococci
PEP	phosphoenolpyruvate
PEtN	phosphoethanolamine
PK/PD	pharmacokinetics/pharmacodynamics
ROS	reactive oxygen species
STR	streptomycin
TDM	therapeutic drug monitoring
UhpT	glucose-6-phosphate transporter
VAN	vancomycin
VRE	vancomycin-resistant enterococci
hVISA/VISA/VRSA	(heterogeneous) vancomycin-intermediate/vancomycin-resistant *S. aureus*
